# Determination of Effective Factors on Power Requirement and Conveying Capacity of a Screw Conveyor under Three Paddy Grain Varieties

**DOI:** 10.1100/2012/136218

**Published:** 2012-04-19

**Authors:** Ezzatollah Askari Asli-Ardeh, Ahmad Mohsenimanesh

**Affiliations:** ^1^Department of Agricultural Machinery, College of Agriculture, University of Mohaghegh Ardabili, Ardabil, Iran; ^2^Agricultural Engineering Research Institute, Shahid Fahmideh Boulevard, P.O. Box 31585-845, Karaj, Iran

## Abstract

An experiment was conducted to investigate the effect of screw speed, inclination angle and variety on the required power, and conveying capacity of a screw conveyor. The experiment was designed with four levels of screw speed (600, 800, 1000, and 1200 rpm), five levels of inclination angle (0, 20, 40, 60, and 80°), and three levels of variety (Alikazemi, Hashemi, and Khazar). The Length, diameter, and pitch of screw were 2, 0.78, and 0.5 m, respectively. The experimental design was a randomized complete block (RCB) with factorial layout. Maximum and minimum power requirements of tested screw conveyor were 99.29 and 81.16 Watt corresponding to conveying capacity of 3.210 and 1.975 ton/hour obtained for khazar and Alikazemi varieties, respectively. The results indicated that as screw inclination angle increased from 0 to 80°, the conveying capacity decreased significantly from 3.581 to 0.932 t/h. It can be concluded that the most conveying capacity was 4.955 t/h at tests with khazar variety and conveyor inclination angle zero degree.

## 1. Introduction

Rice is one of the commonly consumed cereals and food staples for more than half of the world's population. It is an important source of energy, vitamins, mineral elements, and rare amino acids. World rice production increased from 520 million tons in 1990 to 605 million tons in 2004, while Iran's rice production increased from 1.3 million tons in 1980 to 3.4 million tons in 2004 [1].

Application of engineering principles for reducing energy requirement in the form of mechanical and electrical power is necessary to reduce cost of production (http://www.crri.nic.in/Research/Divisions/Engineering.htm). Efficient rice conveying implements are to be used to achieve energy savings. Screw Conveyers (augers) are used to convey free flowing materials such as grain to more difficult fibrous materials such as straw and alfalfa. At gain elevators and on the farmstead, augers are used to move grain to and from storage. In these applications, the conveyor consists of a rotating shaft which carries a helicoid flighting through a stationary tube. Factors affecting capacity include auger dimensions (diameter, auger geometry), shear-plane flighting orientation, auger speed, angle of inclination, commodity being conveyed, and entrance-opening configuration [2].

Nicolai et al. [2] investigated the capacity, volumetric efficiency, and power requirements for a 20 cm and a 25 cm diameter conveyor and operating in a speed range of 250 to 1100 rpm at inclination angles of 13, 20, and 30°. They found that increasing the conveyor speed increased the capacity up to a maximum value, and further increases in speed caused a decrease in capacity. The screw speed for maximum capacity averaged 823 rpm for both 20 cm (8 in) and 25 cm (10 in) diameter of the conveyor. Volumetric efficiency decreased an average of 3% for every 100 rpm increase in conveyor screw speed. Changing the conveyor inclination angle did not affect the volumetric efficiency relative to the screw speed. Power requirements per conveyor screw speed were effected by inclination angle up to 20° but not for inclination angles greater than 20°.

Zareiforoush et al. [3, 4] designed and evaluated a screw conveyor for one rice variety, namely, Hashemi. They found that the specific power requirement of the screw conveyor increased with increasing the screw clearance and screw rotational speed. The net power requirement of the conveyor increased with increasing the screw rotational speed; whilst the value decreased with increasing the screw clearance. As the rotational speed of the screw conveyor increased, the actual volumetric capacity increased up to a maximum value and further increases in speed caused a decrease in capacity. With increasing the screw clearance and screw rotational speed, the volumetric efficiency of the screw conveyor decreased.

In recent times, performance-test procedure develops for screw conveyors designed for different agricultural grains products which characterize conveying capacity, volumetric efficiency, and power requirement. Since grain property variation is wide, especially when considering variety difference, rice cannot be considered to have uniform properties [5]. In other words, variation in rice-milling quality could be as a result of variation in its physical properties [6]. So, mechanical and physical properties of different rice varieties are important in optimum design of machinery for handling. However, a few screw conveyor concern different rice varieties type. Clearly, conveying capacity and requirement power of screw conveyor with respect to conveying materials characteristics are quite different from those of agricultural grains products. There are still improvements to be made for higher accuracy and reliability of screw conveyor. Therefore, an experiment was developed with the following objectives:

1. to evaluate the effects of variety, screw rotational speed, and inclination angle on the requirement power and conveying capacity;
2. to optimize conveying capacity and power requirement of the screw conveyor with respect to the conveying materials characteristics.

## 2. Materials and Methods

An experiment was conducted at Agricultural Machinery Department, Ardabil (Iran), a facility of University of Mohaghegh Ardabili, to investigate the effect of screw speed, inclination angle and variety on the required power and conveying capacity of a screw conveyor. The experiment was designed with four levels of screw speed (600, 800, 1000 and 1200 rpm), five levels of inclination angle (0, 20, 40, 60, and 80°), and three levels of variety (Alikasemi, Hashemi, and Khazar). The experimental design was a randomized complete block (RCB) with factorial layout at four replications. For means comparison, Duncan's Multiple Range Test was used.

**Figure 1:**
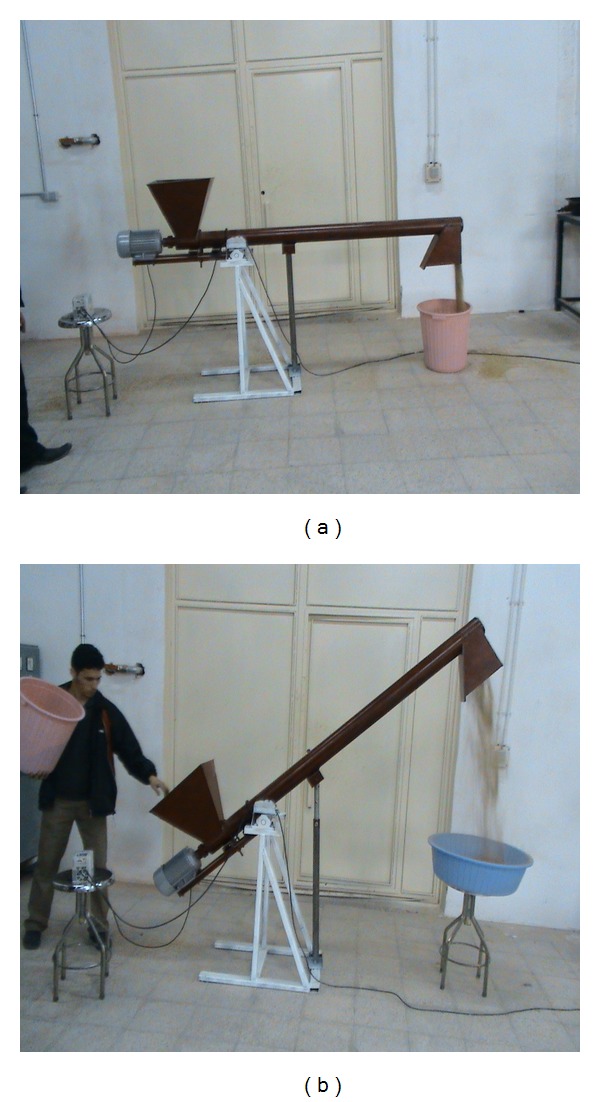
Tested screw conveyor at two conditions of (a) horizontal and (b) inclination angle of 40°.

 The length of screw, screw pitch, and diameter were 2000, 50, and 78 mm, respectively. The clearance between screw and tube is 6 mm, which was developed by Iranian Ashtad cooperative company (Figure 1). A three-phase electromotor (3 HP) was used for driving the screw shaft. Moisture content of paddy Grain was 12 to 13% (w.b.) in all varieties.

An inverter, a rotational speed gauge, and a digital wattmeter were used for continuously determining the variable transmission of screw shaft, the screw rotational speed, and the electromotor electrical power, respectively. The wattmeter was connected to a personal computer for recording and displaying the power data during the tests so that power consumption variations were graphically visible on monitor during the experiments. Each test was started by pouring 30 kg paddy grain into the input section. After a few seconds of unloading grain at outlet section, a container was directed towards the discharge, and then discharged grain mass was weighted. The time of purring of paddy grain into the container was between 10 and 12 seconds. Conveying capacity data was recorded every second for future analyzing.

## 3. Results and Discussion

### 3.1. Power Requirement

The analysis of variance of power requirement and conveying capacity is presented in Table 1. The results showed that the main and interactions' effects of variety, screw rotational speed, and inclination angle were significant (*P* < 1%).

Means comparison results of the main effects revealed that power requirement of Khazar variety was more than others (Table 2). The reason may be due to the differences among the bulk density and friction coefficients of varieties. This is in agreement with the work of Ray [7] and Roberts [8], who found that power requirement varies with physical properties of agricultural grains. By increasing the screw speed from 600 to 1000 rpm, the power requirement was increased and a further increase up to 1200 rpm decreased the power requirement. The reason was that at speeds higher than 1000, centrifugal force prevents axial movement of the grains, and therefore the power requirement is reduced [9]. With increasing of inclination angle up to 40°, the power requirement decreased significantly because of the decrease in the conveying capacity. As inclination angle increased from 40 to 80°, power requirement increased. The reason may be due to paddy grains returning and compacting into the tube. Therefore, friction force and even the amount of grains damage increases. A similar result was obtained by Zareiforoush et al. [5].

**Figure 2:**
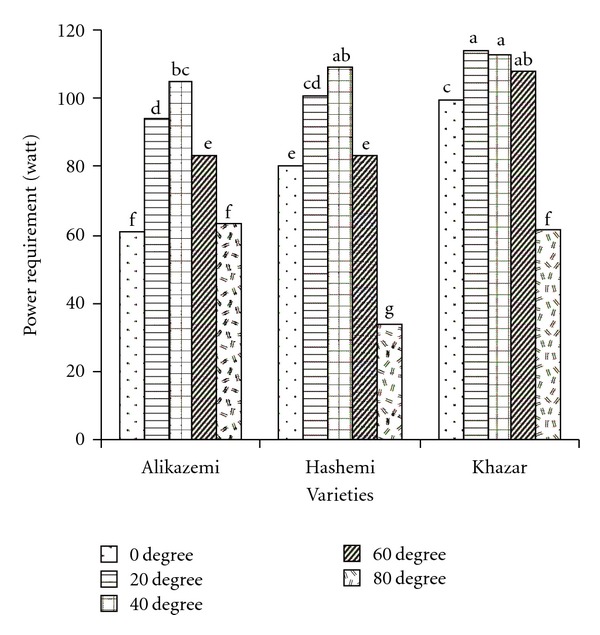
Mean comparison results of interaction effects of variety and inclination angle on power requirement (dissimilar letters show different significantly at probability level %5).

Mean comparison results of interaction effects of variety and inclination angle are shown in Figure 2. With increasing inclination angle from 0 to 40° at tests with Alikazemi and Hashemi varieties, power requirement has been increased significantly. But with further increase in inclination angle up to 80°, power requirement decreased significantly. With increasing inclination angle from 20 to 60° at tests with Khazar variety, power requirement mean was decreased.

**Figure 3:**
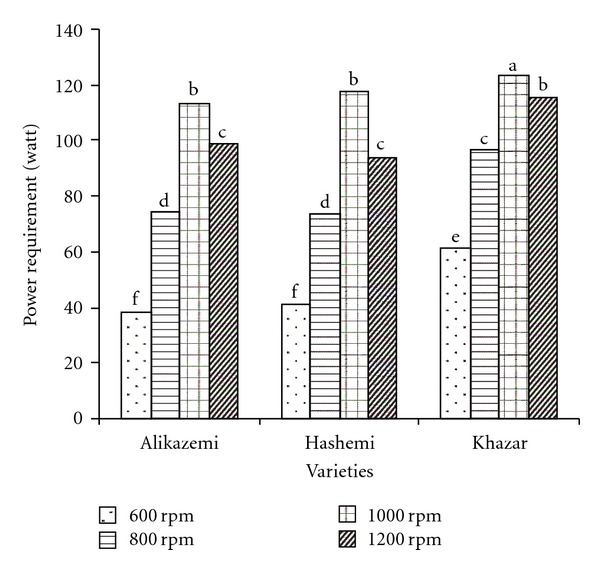
Mean comparison results of interaction effects of variety and screw speed on power requirement (dissimilar letters show different significantly at probability level %5).

Mean comparison results of interaction effects of variety and screw speed are showed in Figure 3. At testes with all varieties, power requirement increased as screw speed increased up to 1000 rpm. The maximum and minimum power requirements were 123.52 and 38.22 W, which was obtained at tests with Khazar variety at screw speed 1000 rpm and with Alikazemi at screw speed 600 rpm, respectively.

**Figure 4:**
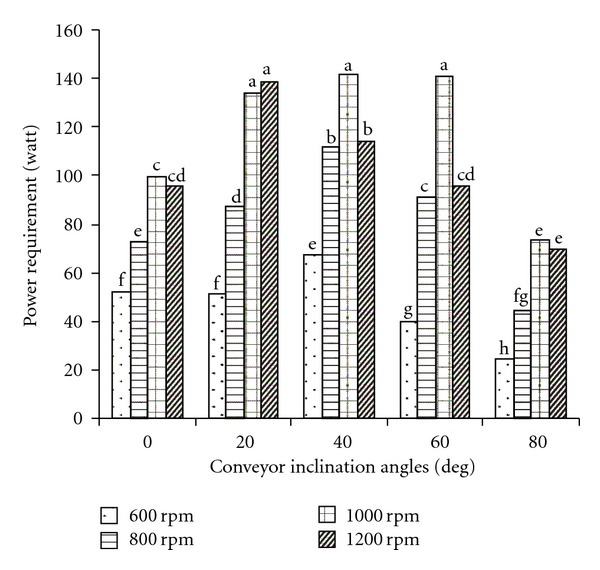
Mean comparison results of interaction effects of inclination angle and screw rotational speed on power requirement (dissimilar letters show different significantly at probability level %5).

Mean comparison results of interaction effects of inclination angle and screw speed are showed in Figure 4. For each angle, power requirement was significantly increased with increasing screw speed up to 1000 rpm. The lowest power requirement was obtained at 80° inclination angle for all levels of the screw rotational speed due to the lowest amount of conveying. A similar result was obtained by Srivastava et al. [9].

 Mean comparison results of interactions between three factors revealed that the most power requirement mean was 156.88 W at tests with Khazar variety, screw speed of 1000 rpm, and inclination angle of 60°.

### 3.2. Conveying Capacity

Analysis of the variance of data obtained from measuring conveying capacity was presented in Table 1. The results showed that the main and interactions effects of variety, screw rotational speed, and inclination angle were significant (*P* < 1%). These results conform to the obtained findings of the researchers in the study of screw conveyor performance [7, 9]. Theoretical capacity of a screw conveyor has confirmed a direct relationship with screw rotational speed. The other factors affecting the conveying capacity are grain bulk density and internal and external friction that may have different values in the paddy grain varieties. By increasing of conveyor inclination angle, weight force of grain mass prevents from the axial movement of grain mass in tube, so the conveying capacity results are reduced. Such trends in the conveying capacity have also been observed in a study of conveyor inclination angle [8].

Means comparison results of variety effects on conveying capacity was significant (*P* < 5%). The highest and lowest conveying capacity was allocated to Khazar and Alikazemi varieties, respectively. By increasing screw speed from 600 to 1000 rpm, conveying capacity was increased significantly and then decreased at test with screw speed 1200 rpm. Similarly, this case had happened for power requirement. By increasing screw inclination angle from 20 to 80°, conveying capacity decreased significantly from 3.352 to 0.932 t/h, respectively. In horizontal state, the screw conveyor had been the greatest amount of 3.581 t/h of conveying capacity. Similar results were obtained by Ray [7] and Srivastava et al. [9].

**Figure 5:**
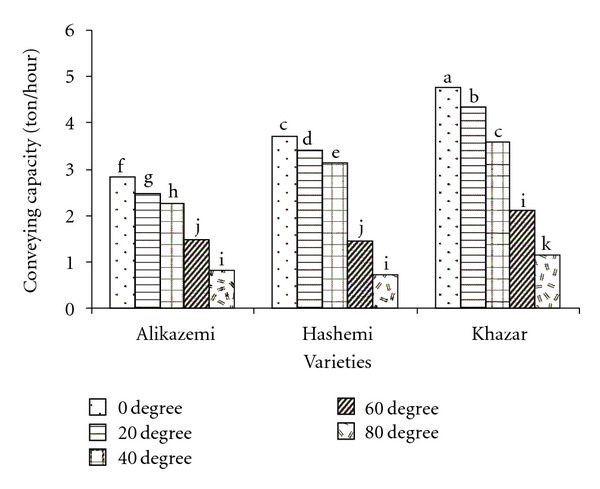
Mean comparison results of interaction effects of variety and conveyor inclination angle on the conveying capacity (dissimilar letters show different significantly at probability level %5).

Mean comparison results of interaction variety and conveyor inclination angle revealed that at tests with all varieties, conveying capacity reduced significantly by changing of conveyor inclination angle from 0 to 80°. In tests with Khazar variety, by changing screw speed from 800 to 1200 rpm, conveying capacity was not significantly changed (Figure 5).

**Figure 6:**
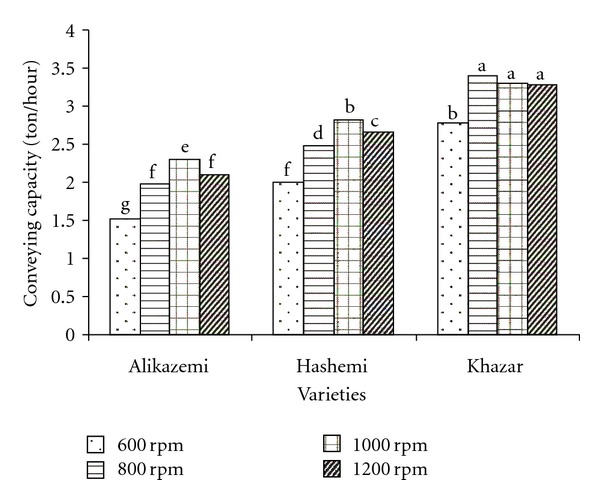
Mean comparison results of interaction effects of variety and screw rotational speed on the conveying capacity (dissimilar letters show different significantly at probability level %5).

Mean comparison results of interaction effects of variety and screw speed were shown in Figure 6. At testes with all varieties, conveying capacity has been increased by increasing screw speed up to 1000 rpm except in Khazar variety. The maximum and minimum conveying capacities were 3.40 and 1.52 t/h, which were obtained at tests with Khazar variety at screw speed 800 rpm and with Alikazemi at screw speed 600 rpm, respectively.

Mean comparison results of interaction effects of three factors showed that the most conveying capacity of 4.955 t/h was obtained at tests with Khazar variety, conveyor inclination angle 0 degree, and screw rotational speed 1000 rpm.

## 4. Conclusions

1. The main effects of variety, conveyor inclination angle, and screw rotational speed and their interactions were significant on power requirement and conveying capacity. Maximum and minimum power requirements of tested screw conveyor were 99.29 and 81.16 Watt corresponding to conveying capacity of 3.210 and 1.975 t/h obtained for khazar and Alikazemi varieties, respectively.
2. The most and least power requirement, and conveying capacity were obtained at screw rotational speeds of 1000 rpm and conveyor inclination angle of 40°, respectively. By increasing the conveyor inclination angle from 0 to 80°, conveying capacity was decreased significantly from 3.581 to 0.932 t/h. Generally, the most conveying capacity was 4.955 t/h at tests with khazar variety and zero inclination angle degree.

## Figures and Tables

**Table 1:** Variance analysis of power requirement and convening capacity.

Variation sources		Power requirement (watt)		Conveying capacity	
	Freedom degree	Square mean	*F* ratio	Square mean	*F* ratio
Replication	3	153	2.930^ns^	0.113	1.274^ns^
Variety (*V*)	2	8635	165.451**	30.88	387.70**
Error	6	52		0.089	
Inclination angle (*α*.)	4	23626	260.637**	68.11	1275.37**
Interactions (*V* × *α*)	8	1762	19.43**	0.053	56.22**
Error	36	90		5.833	
Screw speed (*N*)	3	56790	610.08**	0.203	129.8**
Interactions (*V* × *N*)	6	390	4.196**	0.914	4.62**
Interactions (*α* × *N*)	12	1942	20.86**	0.515	20.34**
Interactions (*V* × *α* × *N*)	24	1575	16.92**	0.045	11.44**
Error	135	93			

Total	239	376298			

**Significant at less than 1% probability levels, ^ns^: not significant.

**Table 2:** The mean comparisons of variety, screw speed, and inclination angle on power requirement and conveying capacity.

	Varieties		Screw speed (rpm)		Inclination angle (degree)	
	Alikazemi	81.16 b	600	46.93 d	0	127.8 a
	Hashemi	81.41 b	800	81.56 c	20	87.28 b
Power requirement (Watt)	Khazar	99.29 a	1000	118.0 a	40	18.59 e
			1200	102.7 b	60	28.11 d
					80	54.42 c

	Alikazemi	1.975 c	600	2.102 c	0	3.581 a
	Hashemi	2.484 b	800	2.621 b	20	3.532 a
Conveying capacity (t/h)	Khazar	3.210 a	1000	2.803 a	40	3.062 b
			1200	2.697 b	60	1.672 c
					80	0.932 d

Dissimilar letters show different significantly at probability level %5.
